# Supplementary material for: Complex‐centric proteome profiling by SEC‐SWATH‐MS
Source: Mol Syst Biol. 2019 Jan 14;15(1):e8438. doi: 10.15252/msb.20188438 (PMC6346213; doi:10.15252/msb.20188438)
Supplement: Supplementary file 8 — Dataset EV7 [file MSB-15-e8438-s008.zip › feature_plots_string/Q9UFF9.pdf]

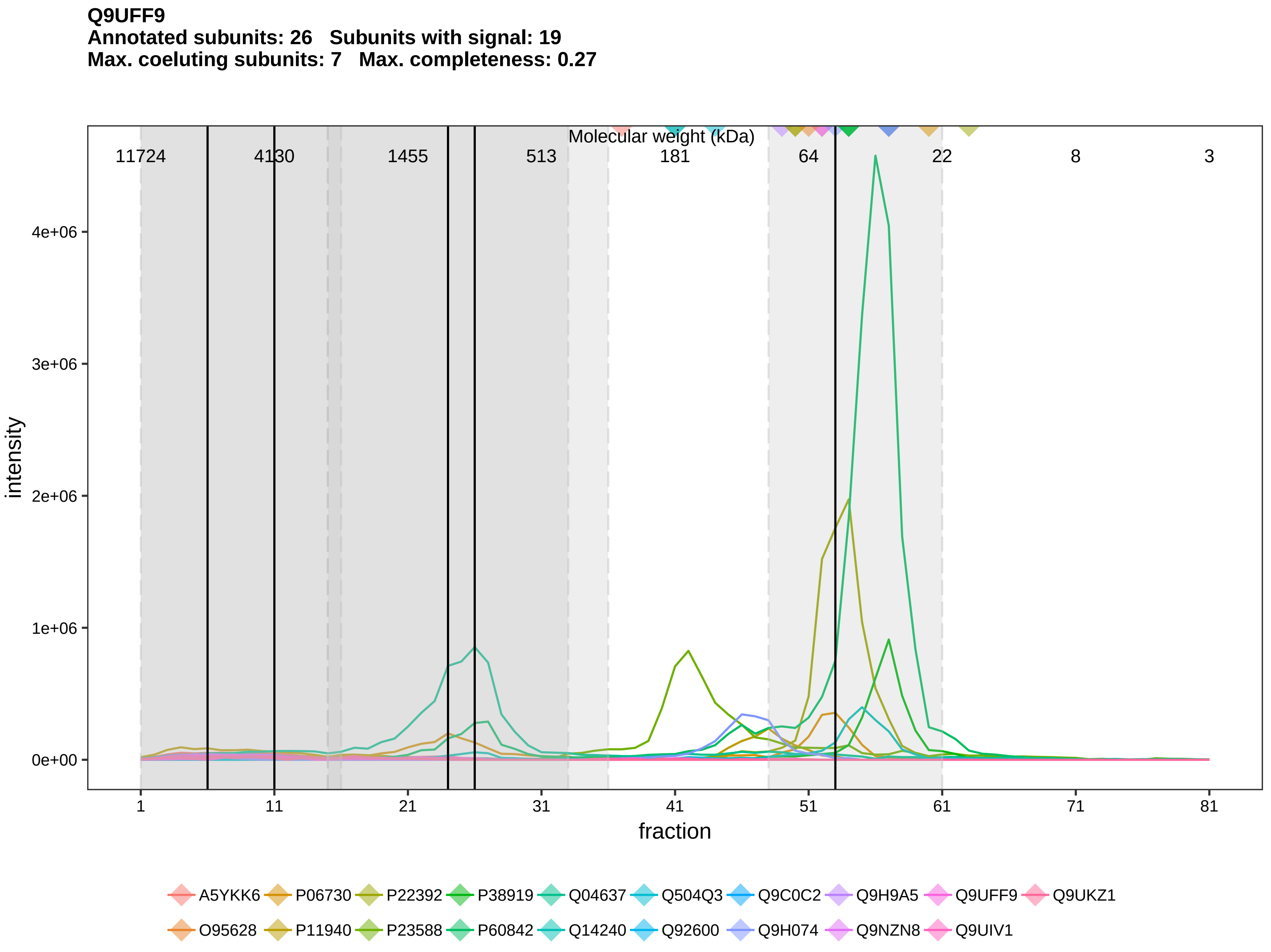

Q9UFF9
Annotated subunits: 26 Subunits with signal: 19
Max. coeluting subunits: 7 Max. completeness: 0.27
Molecular weight (kDa)
11724
4130
1455
513
181
64
22
8
3
intensity
4e+06
3e+06
2e+06
1e+06
0e+00
1
11
21
31
41
51
61
71
81
fraction
A5YKK6
P06730
P22392
P38919
Q04637
Q504Q3
Q9C0C2
Q9H9A5
Q9UFF9
Q9UKZ1
O95628
P11940
P23588
P60842
Q14240
Q92600
Q9H074
Q9NZN8
Q9UIV1
